# *UMOD* polymorphism rs12917707 is not associated with severe or stable IgA nephropathy in a large Caucasian cohort

**DOI:** 10.1186/1471-2369-15-138

**Published:** 2014-08-28

**Authors:** Miriana Dinic, Lidia Ghisdal, Judith Racapé, Lise Thibaudin, Philippe Gatault, Marie Essig, Yann Le Meur, Christian Noël, Guy Touchard, Pierre Merville, Zineb Ajarchouh, Christophe Mariat, Marc Abramowicz, Daniel Abramowicz, Eric Alamartine

**Affiliations:** 1 Nephrology-Renal Transplantation Department, CHU de Saint Etienne & EA3064, GIMAP, Université Jean Monnet, Saint Etienne 42055 Cedex 02, France; 2 Nephrology-Renal Transplantation Department, Universitair Ziekenhuis Antwerp - Université Libre de Bruxelles (ULB), Brussels, Belgium; 3 Department of Biostatistics, School of Public Health, ULB, Brussels, Belgium; 4 Nephrology-Renal Transplantation Department, CHU Tours, Tours, France; 5 Nephrology-Renal Transplantation Department, CHU Limoges, Limoges, France; 6 Nephrology-Renal Transplantation Department, CHU Brest, Brest, France; 7 Nephrology-Renal Transplantation Department, CHRU Lille, Lille, France; 8 Nephrology-Renal Transplantation Department, CHU Poitiers, Poitiers, France; 9 Nephrology-Renal Transplantation Department, CHU Bordeaux, Bordeaux, France; 10 Medical Genetics Department and IRIBHM (Institut de Recherche Interdisciplinaire en Biologie Humaine et Moleculaire), ULB, Brussels, Belgium

## Abstract

**Background:**

Genetic factors are suspected in the pathogenesis of IgA nephropathy, as well as in the course of IgA nephropathy progression towards end stage renal failure. *UMOD* polymorphism rs12917707 is known to associate with end stage renal failure of mixed aetiologies.

**Methods:**

We tested a large cohort of Caucasian patients for association of rs12917707 with IgA nephropathy showing a benign, stable course and with IgA nephropathy that progressed toward end stage renal failure.

**Results:**

No association was observed between either groups, and a non-significant trend was observed for more severe IgA nephropathy with the allele reported to protect against end stage renal failure of mixed aetiologies.

**Conclusion:**

We conclude that *UMOD* is unlikely to play a role in IgA nephropathy pathogenesis nor progression to end stage renal failure, and suggest that *UMOD* effects are restricted to some causes of renal disease, e.g. diabetes or hypertension.

## Background

IgA nephropathy (IgAN) is the most common primary glomerulonephritis worldwide [1]. Thirty percent of IgAN patients will develop End Stage Renal Failure (ESRF) within 20 years after diagnosis requiring dialysis or kidney transplantation.

The pathogenesis of IgA nephropathy is not well understood. A multi-hit mechanism is suggested involving four processes [2]: (i) increase in galactose-deficient circulating IgA1 which alone is not sufficient to trigger the disease, (ii) production of circulating antibodies directed against galactose-deficient IgA1, (iii) formation of pathogenic IgA1-containing circulating immune complexes, (iiii) mesangial deposition of IgA1-containing immune complexes triggering glomerular injury.

Genetic factors are suspected in the pathogenesis of IgA nephropathy: (i) the prevalence of IgA nephropathy varies between ethnic group [3], (ii) familial clustering consistent with autosomal dominant transmission with incomplete penetrance [4-6] (iii) inherited aberrant IgA1 glycosylation in familial and sporadic IgA nephropathy [7]. Several studies have addressed the genetics of IgAN: familial linkage analysis identified IgAN loci with *IgAN1* at chromosome 6q22 emerging as a major locus [8,9]. Genome-wide association studies identified other loci, including the MHC as well as the complement factor H locus and the PSMB gene [10,11].

Finding genetic risk factors for poor outcome of IgAN might allow to identify a high risk subgroup of patients in order to optimize therapeutic strategy, and also to better understand the pathogenesis of IgAN and eventually to characterize novel therapeutic targets.

Genome-wide studies have recently also addressed the genetic variants underlying impaired renal function and Chronic Kidney Disease (CKD) in the general population [12]. Single Nucleotide Polymorphisms (SNPs) in the *UMOD* gene were significantly associated with CKD and increased serum creatinine. The minor T allele was associated with a 20% reduced risk of CKD. The *UMOD* gene is located at chromosome 16p12 and encodes the renal-specific protein uromodulin, or Tamm-Horsfall protein, which is the most abundant protein in the urine of healthy individuals. The function of uromodulin is still unclear but it may confer protection against inflammation and infection. Rare, highly penetrant mutations in the *UMOD* gene are known to cause medullary cystic kidney diease or familial juvenile hyperuricemic nephropathy. The *UMOD* gene is transcribed exclusively in renal tubular cells of the thick ascending limb of the loop of Henle. These findings hence suggest a common mechanism for CKD pathogenesis localized at the nephron’s loop of Henle with an important role of uromodulin.

In addition, associations were found between *UMOD* and predictive factors of IgAN progression such as hypertension and tubular atrophy and interstitial atrophy. First, a GWAS identified a locus in the 5′ region of UMOD gene whose minor allele is associated with a lower risk of hypertension [13]. This result suggests the role of uromodulin in blood pressure regulation. Hypertension is known as IgAN factors predicting and accelerating progression to end-stage renal disease [14]. Second, a study found that urinary uromodulin level was associated with tubulointerstitial lesions in IgAN [15] which is known as a predicting factor with the OXFORD-MEST scoring [16]. These findings suggest that *UMOD* gene may be involved in the pathogenic process of renal disease.

Here, we tested the hypothesis that *UMOD* polymorphism rs12917707 is associated with severe outcome in IgA nephropathy. In a large cohort of Caucasian patients, we compared the frequency of the T allele in the following groups: stable IgAN, IgAN associated with severe outcome, and healthy controls.

## Methods

### Patients

**Figure 1:**
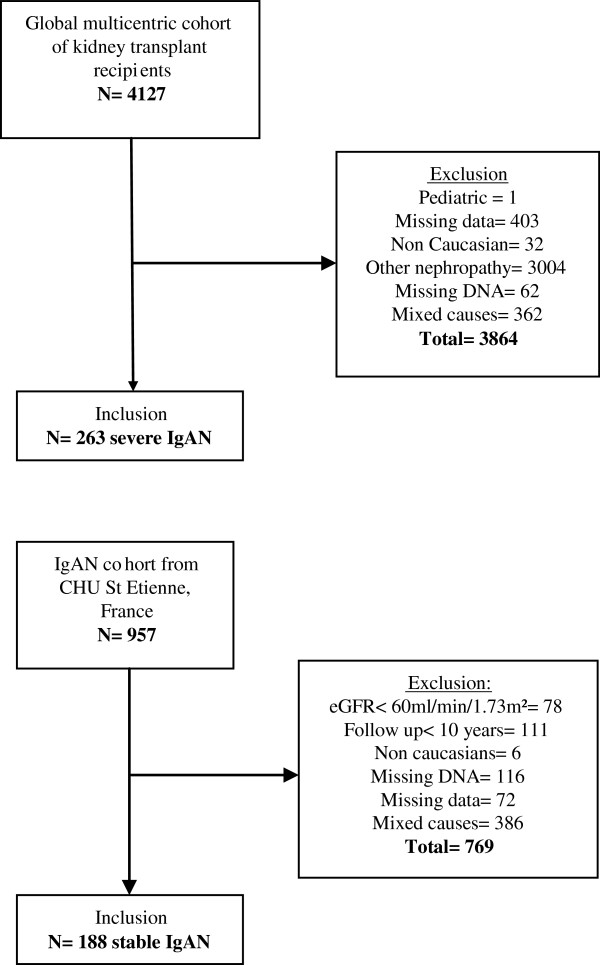
Inclusion IgAN cases.

We constituted two groups of patients based on the disease phenotype. The first group was composed of IgAN cases with severe outcome. The inclusion criteria consisted of adult Caucasian patients with a biopsy-proven IgAN and a poor outcome as indicated by terminal renal failure that required kidney transplantation. This population came from a franco-belgian consortium of 8 European renal transplant centers (ULB-Hôpital Erasme- Brussels, Belgium, CHU Tours, CHU Limoges, CHU Brest, CHU Saint Etienne, CHRU Lille, CHU Poitiers and CHU Bordeaux, France). DNA samples and clinical data came from a global cohort of 4127 renal allograft recipients. This first group included 263 patients with severe IgAN. A second group was composed of stable cases of IgAN with no CKD whose inclusion criteria were: Caucasian adult patients with biopsy-proven IgAN diagnosed since at least 10 years, with eGFR > 60 ml/min/1.73 m^2^. This group of 188 patients came from the cohort of CHU Saint Etienne, France. This biological collection has been declared to the French Health Ministry. A third, control group, consisted of Caucasian healthy volunteers from the control cohort of ULB-Hôpital Erasme. Data and DNAs were centralized in ULB-Hôpital Erasme. Informed consents were obtained from each participants and the study followed good clinical practices. The study was realized with approval from the local ethical committee of ULB-Hôpital Erasme- Brussels, Belgium and CHU de Saint Etienne. This study is in compliance with the Helsinki Declaration. Figure 1 represents the cases flow chart.

### Genotyping analyses

For this project involving only one SNP and a large population, the TaqMan assay was the preferred technology as it has high throughput and is highly accurate, precise, time-efficient, and cost-effective.

TaqMan SNP genotyping assays were used for SNP genotyping (Applied Biosystem). PCRs were performed in 96-well plates with a final reaction volume of 15 μl, containing 10 ng of genomic DNA, 7.5 μl of TaqMan Genotyping Master Mix (Applied Biosystem), 0.75 μl of TaqMan SNP Genotyping assay mix (containing rs12917707 specific primers and TaqMan MGB probes) and 1.75 μl of water. Two negative template controls were tested in each plate. PCR steps were as follow: 95°C for 10 min followed by 40 cycles of 92°C for 15 s and 60°C for 60 s with an automatic post-read run giving patient genotyping. All the PCRs were performed on a 7500 Fast Real-time PCR system (Applied Biosystem).

### Statistic analyses

We tested the hypothesis that *UMOD* polymorphism rs12917707 is associated with severe outcome in IgAN. The primary objective was to evaluate the association of rs12917707 in *UMOD* gene with severe IgAN. We tested the null hypothesis of no association between this SNP and severe IgAN. A sample size calculation was made for unmatched case–control study. A study comparing 263 cases and 338 controls has 80% power to detect a protective effect of T allele (primary endpoint) with an OR = 0.5, with an alpha error of 5% (ref: http://www.openepi.com ). We compared the genotype distributions (GG, GT and TT) and frequencies of T and G alleles between the 3 groups and in a second time we realized pairwise comparisons (severe cases versus controls; stable cases versus controls; severe cases versus stable cases). Categorical data were compared using the χ^2^ test. We confirmed that the genotypes distributions followed the Hardy-Weinberg equilibrium using the χ^2^ test for the minor allele for the global cohort genotyped (severe IgAN, stable IgAN and healthy volunteers).

## Results

Two hundred and sixty three patients with severe IgAN, one hundred and eighty eight patients with stable IgAN and three hundred and forty five healthy control subjects were included in the analysis. The mean age of patients with severe IgAN was 50.68 +/- 14.39 years at the time of transplantation with a male/female sex ratio of 2.6. The mean age of stable IgAN cases was 28.04 +/- 6.24 years at the time of diagnosis; the mean duration of disease at last follow up was 24.21+/- 18.96 years and with a male/female sex ratio of 2.3. The main characteristics at last follow up are shown in Table 1.

**Table 1:** Stable IgAN characteristics at last follow up

Age at diagnostic, years (mean ± DS)	28.03 ± 6.25
Age at follow up, years (mean ± DS)	47.82 ± 12.7
Follow up duration, years (mean ± DS)	24.2 ± 18.96
Proteinuria at follow up, g/d (mean ± DS)	0.39 ± 0.13
SBP at follow up, mm Hg (mean ± DS)	128.76 ± 16.3
DBP at follow up, mm Hg (mean, ± DS)	78.98 ± 2.83
Serum creatinine at follow up, μmol/l (mean ± DS)	84.59 ± 43.84
eGFR, ml/min/1.73 ml^2^ (mean ± DS)	86.32 ± 38.18

*SBP* systolic Blood Pressure, *DBP* Diastolic Blood Pressure, *eGFR* estimated Glomerular Filtration (MDRD equation).

The G and T allele frequencies of *UMOD* polymorphism rs12917707 were 82.7% and 17.7% respectively in the severe IgAN group, 83.8% and 16.2% in the stable IgAN group, and 83.6% and 16.4% in the control cohort. These frequencies did not significantly differ (p = 0.69 for the comparison between the 3 groups, p = 0.58 for comparison between severe cases versus stable cases and between severe cases and healthy controls, p = 0.84 for comparison between stable cases and healthy controls).

In the severe IgAN group, the frequencies of rs12917707 genotypes GG, GT and TT were 68.4%, 27.8% and 3.8% respectively. These frequencies did not significantly differ from those of the control population (70.7%, 26.1% and 3.2% respectively) in any inheritance model (co-dominant, dominant or recessive). The same was true when comparing genotypes in the stable IgAN group against the healthy control group. These results summarized in Table 2. For the global cohort (n = 796), minor allelic frequency (MAF) of rs12917707 was 0.17 and genotypes fitted the Hardy-Weinberg equilibrium (p = 0.49).

**Table 2:** Allele frequencies and genotypes distribution among severe cases of IgAN, stable cases of IgAN and healthy volunteers

**Genotype and allele of rs 12917707**	**Severe cases (n, %) N = 263**	**Stable cases (n, %) N = 188**	**Controls (n, %) N = 345**	P*	P**	P***	P****
**GG**	180(68.4)	133(70.7)	245(69.9)	0.91	0.69	0.88	0.85
**GT**	73(27.8)	49(26.1)	95(27.5)				
**TT**	10(3.8)	6(3.2)	9(2.6)				
**G**	433(82.3)	315(83.8)	577(83.6)	0.79	0.60	1.00	0.63
**T**	93(17.7)	61(16.2)	113(16.4)				

P* refers to comparison between the 3 groups; p** refers to comparison between severe cases and controls; p*** refers to comparison between stable cases and controls; p**** refers to comparison between severe and stable cases.

## Discussion

In this study, we compared *UMOD* polymorphism rs12917707 in a large cohort of Caucasian patients, with either severe IgAN (end-stage renal failure requiring renal transplantation), or stable IgAN (eGFR > 60 ml/min/1.73 m^2^ for at least ten years since diagnosis), and healthy controls.

Our main result is the lack of significant difference in allele and genotype proportions between these three groups.

The similar *UMOD* genotype frequencies between healthy controls and severe IgAN patients indicate that *UMOD* is unlikely to be involved in IgAN pathogenesis. The similar *UMOD* genotype frequencies between stable IgAN and severe IgAN further indicate that *UMOD* is unlikely to play a role in the progression of IgAN towards renal failure. Furthermore, although our results failed to reach statistical significance, minor T allele frequency was higher in the group with severe IgAN as compared with the stable IgAN group. Therefore the minor T allele doesn’t seem to be protective against CKD progression in IgAN.

Our findings are divergent from GWAS performed to identify loci associated with CKD susceptibility or GFR (quantitative trait). Several explanations might contribute to these differences.

First, the major difference is the type of population included. We have enrolled patients selected for a specific disease (IgAN), whereas, the main GWAS, followed by meta-analysis enrolled mainly population-based cohorts unselected for a trait or a disease [12,17,18]. In such cohorts, the proportion of CKD cases with hypertension and/or type 2 diabetes was high [12,19]. It has been shown in a quantitative-trait GWAS that the effect of *UMOD* polymorphism on SCr increases substantially with both age and number of comorbid diseases (hypertension, diabetes mellitus, atherosclerosis and heart failure) [20]. This GWAS showed even no impact of *UMOD* polymorphism in patients <50 years, of note, the mean age of our cohort with severe IgAN was 50.68 +/- 14.39 years at the time of transplantation and the mean age of stable IgAN cases was 28.04 +/- 12.17 years at the time of diagnosis. Padmanabhan et al. [13] corroborated the hypothesis of *UMOD* gene’s implication in individuals having common risk factors by performing a GWAS which identified *UMOD* polymorphism rs13333226 whose minor G allele was associated with a lower risk of hypertension and higher eGFR. It was also associated with a lower risk of cardiovascular events after adjusting for age, sex, BMI and smoking status. Furthermore, it seems that *UMOD* gene interacts with sodium excretion. The association between *UMOD* in unselected populations and the absence of association observed in our IgA-selected population supports the hypothesis that *UMOD* might increase susceptibility of CKD in individuals having common risk factors (hypertension or diabetes). The other hypothesis to consider is that IgAN progression might not follow common pathways leading to ESRF.

Second, we tested the association in patients with stage V CKD requiring transplantation. GWAS detecting an association between *UMOD* and CKD defined as an eGFR < 60 ml/min/1.73 m^2^ including a minority of stage V CKD cases with in this cohort a mean eGFR around 80 ml/min/1.73 m^2^ and almost 13.6% of patients with an eGFR < 60 ml/min/1.73 m^2^. We also tested the association in patients with stable IgAN defined as patients whose IgAN was diagnosed since at least 10 years, with still an eGFR > 60 ml/min/1.73 m^2^. The lack of association observed in this group may be explained by the definition of CKD given in GWAS as an eGFR < 60 ml/min/1.73 m^2^ which doesn’t concur with our stable group. A borderline protective effect of the minor allele in the risk of ESRD was shown in two large case–control studies [21,22]. In the first study, the authors did not find an association between the SNP and aetiology of ESRD. The first cohort included 7.8% of IgAN [21], while the cause of ESRD was not described in the second [22]. The observed unspecific and borderline effect size of *UMOD* polymorphism in these two studies argues again for a universal, non-specific effect of the SNP on renal function decline irrespectively of underlying primary disease.

Our study has several limitations. Our study is underpowered to detect a small effect size, as observed in GWAS based on unselected population. The effect size of the protective allele of *UMOD* polymorphism is modest with regards to CKD risk in large GWAS. We have hypotheses that the OR for the protective allele would be larger in our group of ESRD than the OR observed in GWAS, including mostly stage I, II or III CKD cases. Our sample size had the theoretical power to detect a protective effect of T allele with an OR = 0.5. A sample size of 2064 cases and 2662 controls would have detect a OR of 0.8 for a protective effect of minor allele (effect size found in CKD-GWAS) with a power of 80% and alpha error of 5% (http://www.openepi.com). Second we used a single candidate gene approach to evaluate the genetic susceptibility of a complex disease. The hypothesis-free approach of GWAS is the best way to find out common variants associated with complex diseases. Here we tested the hypothesis that a major CKD-associated SNP from GWAS might be involved in severe IgAN with a strong effect size. The role of less frequent variants with a strong effect size in complex diseases is now recognized [23,24]. The search for such variants is a complementary unbiased approach in the study of complex diseases. Our group is therefore performing whole exome sequencing in patients with severe IgAN, in order to find out rare variants strongly associated with this complex disease.

Some may argue that *UMOD* is likely not associated with IgA nephropathy as GWAS did not demonstrate an association between IgAN and common variants of *UMOD*[10,11]. However, such GWAS included various stage of biopsy-proven IgAN, such as the GWAS of Gharavi et al. including a majority of stage I and II CKD IgAN cases from Asian and Caucasian ancestry [10]. These large studies are underpowered to detect a large effect size of *UMOD* variant rs12917707 in the subgroup of Caucasian patients with severe IgAN (CKD V).

## Conclusion

Our study in Caucasian patients didn’t find an association of *UMOD* gene variant rs12917707 with IgAN, nor with the progression of IgAN towards end stage renal failure, in spite of the known association of this variant with chronic kidney disease of mixed aetiologies. Our findings are consistent with *UMOD* playing a role in kidney diseases caused by some risk factors like diabetes or hypertension, but not by IgAN.

## Competing interests

The authors declare that they have no competing interests.

## Authors’ contributions

MD carried out the molecular genetic study, carried out the genotyping assays, participated in the design of the study, performed the statistical analysis and drafted the manuscript. LG carried out the molecular genetic study, participated in the design of the study, performed the statistical analysis and drafted the manuscript. JR performed the statistical analysis. LT, PG, ME, YLM, CN, GT and PM contributed to the creation of DNA collection. ZA contributed to the creation of DNA collection and carried out the genotyping assays. CM contributed to the creation of DNA collection and drafted the manuscript. DA conceived of the study, participated in its design and coordination, and drafted the manuscript. MA and EA conceived of the study, participated in its design and coordination, and drafted the manuscript. All authors read and approved the final manuscript.

## Pre-publication history

The pre-publication history for this paper can be accessed here:

http://www.biomedcentral.com/1471-2369/15/138/prepub

## References

[B1] D’Amico G The commonest glomerulonephritis in the world: IgA nephropathy Q J Med 1987 64 245 709 727 3329736

[B2] Suzuki H Kiryluk K Novak J Moldoveanu Z Herr AB Renfrow MB Wyatt RJ Scolari F Mestecky J Gharavi AG Julian BA The pathophysiology of IgA nephropathy J Am Soc Nephrol 2011 22 10 1795 1803 10.1681/ASN.2011050464 21949093 PMC3892742

[B3] Hall YN Fuentes EF Chertow GM Olson JL Race/ethnicity and disease severity in IgA nephropathy BMC Nephrol 2004 5 10 10.1186/1471-2369-5-10 15341669 PMC517500

[B4] Julian BA Quiggins PA Thompson JS Woodford SY Gleason K Wyatt RJ Familial IgA nephropathy. Evidence of an inherited mechanism of disease N Engl J Med 1985 312 4 202 208 10.1056/NEJM198501243120403 3855328

[B5] Levy M Familial cases of Berger’s disease or of Berger’s disease and rheumatoid purpura. Cooperative study of the Société Française de Néphrologie Nephrologie 1989 10 4 175 182 2699008

[B6] Scolari F Amoroso A Savoldi S Mazzola G Prati E Valzorio B Viola BF Nicola B Movilli E Sandrini M Campanini M Maiorca R Familial clustering of IgA nephropathy: further evidence in an Italian population Am J Kidney Dis 1999 33 5 857 865 10.1016/S0272-6386(99)70417-8 10213640

[B7] Gharavi AG Moldoveanu Z Wyatt RJ Barker CV Woodford SY Lifton RP Mestecky J Novak J Julian BA Aberrant IgA1 glycosylation is inherited in familial and sporadic IgA nephropathy J Am Soc Nephrol 2008 19 5 1008 1014 10.1681/ASN.2007091052 18272841 PMC2386728

[B8] Gharavi AG Yan Y Scolari F Schena FP Frasca GM Ghiggeri GM Cooper K Amoroso A Viola BF Battini G Caridi G Canova C Farhi A Subramanian V Nelson-Williams C Woodford S Julian BA Wyatt RJ Lifton RP IgA nephropathy, the most common cause of glomerulonephritis, is linked to 6q22-23 Nat Genet 2000 26 3 354 357 10.1038/81677 11062479

[B9] Bisceglia L Cerullo G Forabosco P Torres DD Scolari F Di Perna M Foramitti M Amoroso A Bertok S Floege J Mertens PR Zerres K Alexopoulos E Kirmizis D Ermelinda M Zelante L Schena FP Genetic heterogeneity in Italian families with IgA nephropathy: suggestive linkage for two novel IgA nephropathy loci Am J Hum Genet 2006 79 6 1130 1134 10.1086/510135 17186473 PMC1698717

[B10] Gharavi AG Kiryluk K Choi M Li Y Hou P Xie J Sanna-Cherchi S Men CJ Julian BA Wyatt RJ Novak J He JC Wang H Lv J Zhu L Wang W Wang Z Yasuno K Gunel M Mane S Umlauf S Tikhonova I Beerman I Savoldi S Magistroni R Ghiggeri GM Bodria M Lugani F Ravani P Ponticelli C Genome-wide association study identifies susceptibility loci for IgA nephropathy Nat Genet 2011 43 4 321 327 10.1038/ng.787 21399633 PMC3412515

[B11] Feehally J Farrall M Boland A Gale DP Gut I Heath S Kumar A Peden JF Maxwell PH Morris DL Padmanabhan S Vyse TJ Zawadzka A Rees AJ Lathrop M Ratcliffe PJ HLA has strongest association with IgA nephropathy in genome-wide analysis J Am Soc Nephrol 2010 21 10 1791 1797 10.1681/ASN.2010010076 20595679 PMC3013538

[B12] Köttgen A Glazer NL Dehghan A Hwang S-J Katz R Li M Yang Q Gudnason V Launer LJ Harris TB Smith AV Arking DE Astor BC Boerwinkle E Ehret GB Ruczinski I Scharpf RB Chen Y-DI De Boer IH Haritunians T Lumley T Sarnak M Siscovick D Benjamin EJ Levy D Upadhyay A Aulchenko YS Hofman A Rivadeneira F Uitterlinden AG Multiple loci associated with indices of renal function and chronic kidney disease Nat Genet 2009 41 6 712 717 10.1038/ng.377 19430482 PMC3039280

[B13] Padmanabhan S Melander O Johnson T Di Blasio AM Lee WK Gentilini D Hastie CE Menni C Monti MC Delles C Laing S Corso B Navis G Kwakernaak AJ van der Harst P Bochud M Maillard M Burnier M Hedner T Kjeldsen S Wahlstrand B Sjögren M Fava C Montagnana M Danese E Torffvit O Hedblad B Snieder H Connell JMC Brown M Genome-wide association study of blood pressure extremes identifies variant near UMOD associated with hypertension PLoS Genet 2010 6 10 e1001177 10.1371/journal.pgen.1001177 21082022 PMC2965757

[B14] Coppo R D’Amico G Factors predicting progression of IgA nephropathies J Nephrol 2005 18 5 503 512 16299675

[B15] Zhou J Chen Y Liu Y Shi S Wang S Li X Zhang H Wang H Urinary uromodulin excretion predicts progression of chronic kidney disease resulting from IgA nephropathy PLoS One 2013 8 8 e71023 10.1371/journal.pone.0071023 23990922 PMC3750049

[B16] Coppo R Cattran D Roberts Ian SD Troyanov S Camilla R Cook T Feehally J The new Oxford clinico-pathological classification of IgA nephropathy Prilozi 2010 31 1 241 248 20693944

[B17] Chambers JC Zhang W Lord GM van der Harst P Lawlor DA Sehmi JS Gale DP Wass MN Ahmadi KR Bakker SJL Beckmann J Bilo HJG Bochud M Brown MJ Caulfield MJ Connell JMC Cook HT Cotlarciuc I Davey Smith G De Silva R Deng G Devuyst O Dikkeschei LD Dimkovic N Dockrell M Dominiczak A Ebrahim S Eggermann T Farrall M Ferrucci L Genetic loci influencing kidney function and chronic kidney disease Nat Genet 2010 42 5 373 375 10.1038/ng.566 20383145 PMC3748585

[B18] Okada Y Sim X Go MJ Wu J-Y Gu D Takeuchi F Takahashi A Maeda S Tsunoda T Chen P Lim S-C Wong T-Y Liu J Young TL Aung T Seielstad M Teo Y-Y Kim YJ Lee J-Y Han B-G Kang D Chen C-H Tsai F-J Chang L-C Fann S-JC Mei H Rao DC Hixson JE Chen S Katsuya T Meta-analysis identifies multiple loci associated with kidney function-related traits in east Asian populations Nat Genet 2012 44 8 904 909 10.1038/ng.2352 22797727 PMC4737645

[B19] Köttgen A Pattaro C Böger CA Fuchsberger C Olden M Glazer NL Parsa A Gao X Yang Q Smith AV O’Connell JR Li M Schmidt H Tanaka T Isaacs A Ketkar S Hwang S-J Johnson AD Dehghan A Teumer A Paré G Atkinson EJ Zeller T Lohman K Cornelis MC Probst-Hensch NM Kronenberg F Tönjes A Hayward C Aspelund T New loci associated with kidney function and chronic kidney disease Nat Genet 2010 42 5 376 384 10.1038/ng.568 20383146 PMC2997674

[B20] Gudbjartsson DF Holm H Indridason OS Thorleifsson G Edvardsson V Sulem P De Vegt F D’ Ancona FCH Den Heijer M Wetzels JFM Franzson L Rafnar T Kristjansson K Bjornsdottir US Eyjolfsson GI Kiemeney LA Kong A Palsson R Thorsteinsdottir U Stefansson K Association of variants at UMOD with chronic kidney disease and kidney stones-role of age and comorbid diseases PLoS Genet 2010 6 7 e1001039 10.1371/journal.pgen.1001039 20686651 PMC2912386

[B21] Reznichenko A Böger CA Snieder H van den Born J De Borst MH Damman J Van Dijk MCRF Van Goor H Hepkema BG Hillebrands J-L Leuvenink HGD Niesing J Bakker SJL Seelen M Navis G UMOD as a susceptibility gene for end-stage renal disease BMC Med Genet 2012 13 78 22947327 10.1186/1471-2350-13-78 PMC3495046

[B22] Böger CA Gorski M Li M Hoffmann MM Huang C Yang Q Teumer A Krane V O’Seaghdha CM Kutalik Z Wichmann H-E Haak T Boes E Coassin S Coresh J Kollerits B Haun M Paulweber B Köttgen A Li G Shlipak MG Powe N Hwang S-J Dehghan A Rivadeneira F Uitterlinden A Hofman A Beckmann JS Krämer BK Witteman J Association of eGFR-Related Loci Identified by GWAS with Incident CKD and ESRD PLoS Genet 2011 7 9 e1002292 10.1371/journal.pgen.1002292 21980298 PMC3183079

[B23] Manolio TA Collins FS Cox NJ Goldstein DB Hindorff LA Hunter DJ McCarthy MI Ramos EM Cardon LR Chakravarti A Cho JH Guttmacher AE Kong A Kruglyak L Mardis E Rotimi CN Slatkin M Valle D Whittemore AS Boehnke M Clark AG Eichler EE Gibson G Haines JL Mackay TFC McCarroll SA Visscher PM Finding the missing heritability of complex diseases Nature 2009 461 7265 747 753 10.1038/nature08494 19812666 PMC2831613

[B24] Kiezun A Garimella K Do R Stitziel NO Neale BM McLaren PJ Gupta N Sklar P Sullivan PF Moran JL Hultman CM Lichtenstein P Magnusson P Lehner T Shugart YY Price AL De Bakker PIW Purcell SM Sunyaev SR Exome sequencing and the genetic basis of complex traits Nat Genet 2012 44 6 623 630 10.1038/ng.2303 22641211 PMC3727622

